# Enhanced Connectivity of Thalamo-Cortical Networks in First-Episode, Treatment-Naive Somatization Disorder

**DOI:** 10.3389/fpsyt.2020.555836

**Published:** 2020-09-11

**Authors:** Jin Zhao, Qinji Su, Feng Liu, Zhikun Zhang, Ru Yang, Wenbin Guo, Jingping Zhao

**Affiliations:** ^1^ National Clinical Research Center for Mental Disorders, and Department of Psychiatry, The Second Xiangya Hospital of Central South University, Changsha, China; ^2^ Department of Psychiatry, Henan Mental Hospital, Second Affiliated Hospital of Xinxiang Medical University, Xinxiang, China; ^3^ Mental Health Center, the Second Affiliated Hospital of Guangxi Medical University, Nanning, China; ^4^ Department of Radiology, Tianjin Medical University General Hospital, Tianjin, China; ^5^ Department of Radiology, The Second Xiangya Hospital of Central South University, Changsha, China; ^6^ Department of Psychiatry, The Third People’s Hospital of Foshan, Foshan, China

**Keywords:** thalamus, functional connectivity, somatization disorder, anterior/middle cingulum, motor/sensory cortex

## Abstract

**Background:**

Dysfunctions of the thalamus and its projections to cortical cortices have been implicated in patient with somatization disorder (SD). However, changes in the anatomical specificity of thalamo-cortical functional connectivity (FC) in SD remain unclear.

**Methods:**

Resting-state fMRI scans were collected in 25 first-episode, drug-naive patients with SD, as well as 28 sex-, age-, and education-matched healthy controls. We parcellated the thalamus with seven predefined regions of interest (ROIs) and used them as seeds to map whole-brain FC. Correlation analysis was conducted in the patients.

**Results:**

We found an increased pattern of thalamic ROI-cortex connectivity in patients with SD. Patients with SD demonstrated enhanced thalamic connectivity to the bilateral anterior/middle cingulum, motor/sensory cortex, visual cortex, and auditory cortex. A significantly negative correlation was found between the right occipital thalamic ROI to the anterior cingulum and EPQ extraversion scores (*r*=0.404, *p*=0.045) after the Benjamini-Hochberg correction.

**Conclusions:**

This study demonstrates that anatomical specificity of enhanced thalamo-cortical FCs exists in first-episode, drug-naive patients with SD. These findings further highlight the importance of the thalamic subregions in the pathophysiology of SD.

## Introduction

Somatization disorder (SD) is a common mental disease with a prevalence of 4% to 7% in general individuals (1). Patients with SD are always stressed because of their unexplained physical symptoms (e.g. pain, gastrointestinal distress, and pseudoneurological symptoms) and repeated hospital visits. SD places a huge burden on patients and families, and continues to lead patients experiencing a low life quality and high emotional stress. Although many studies have been conducted on SD, the pathophysiology of SD remains unclear.

With the progress of neuroimaging techniques, MRI has become a promising tool for observation of brain structure, activity, and connectivity of SD. Structural MRI studies show that patients with SD have increased gray matter volume in the caudate nuclei (2), but reduced gray volumes in the amygdala and pituitary (3, 4). Zhao et al. found increased white matter volume in the right inferior frontal gyrus and decreased white matter volume in the left inferior longitudinal fasciculus in patients with SD (5). Functional MRI studies also report altered activity and connectivity in patients with SD. Fayed et al. used spectroscopy to evaluate resting-state glutamate and glutamine levels in patients with fibromyalgia, patients with SD and healthy controls, and found that patients with SD showed enhanced glutamate and glutamine levels in the posterior cingulate cortex (6). Moreover, increased activity in the bilateral superior medial prefrontal cortex and left precuneus have been observed in patients with SD by Su et al.(7), who also found enhanced FC strength in the inferior temporal gyrus (ITG) in SD (8). Li et al. found increased/decreased bidirectional corticolimbic connectivity and bidirectional cortico-cerebellar and limbic-cerebellar connectivity in patients with SD (9). Despite these findings supporting abnormal FCs in patients with SD, the effects of important FCs (such as thalamo-cortical FC) on the pathophysiology of SD remain unclear.

The thalamus, a relay station that organizes information routes within the cortex, may play an important role in the FC network of the brain. Previous studies have shown disrupted thalamo-cortical FC in mental diseases. For example, deformed thalamic shape (10), reduced regional thalamic volumes (11), altered correlational patterns between thalamic and cortical volumes (12, 13), and abnormal functional connectivity (14) were reported in schizophrenia. Wang et al. found increased and reduced thalamic FCs in patients with schizophrenia (15). Skåtun et al. conducted thalamo-cortical FC in schizophrenia and found that one increased thalamic-sensory connectivity and eight reductions with frontal and posterior areas in schizophrenia (16). Greicius et al. observed increased FC between the thalamus and the default mode network in major depressive disorder (MDD) (17). However, changes in the thalamo-cortical networks in patients with SD remain unknown.

The thalamus comprises many distinct nuclei and reciprocal topographically organized fibers that connect with the sensory, motor, limbic, and cognitive regions of the cortex (18, 19). For example, the ventral lateral and ventral posteriolateral portions of the thalamus connect to the motor and somatosensory areas, whereas the anterior and dorsomedial areas of the thalamus are linked to the prefrontal cortex (20). De Greck et al. conducted a reward task-fMRI, and patients with acute somatoform disorder patients showed hemodynamic changes in the right ventroposterior thalamus (21). Kang et al. reported enhanced FC between the thalamus and the primary somatosensory cortex in MDD (22). In addition, the thalamus exhibited discrimination-related activation during spatial or non-spatial discrimination of pain stimuli (23), and dynamic analysis identified the thalamus as a critical region in the prediction of pain intensity (24).Thus, topographical division of the FC between the thalamus and the cortex may improve the possibility that mental diseases (such as SD) can be characterized by specific dysfunction connectivity in the thalamic subdivision (or cortical regions).

Based on a previous study, the thalamus can be segmented as bilateral seven subregions, including primary motor, sensory, occipital, pre-frontal, pre-moto, posterior parietal, and temporal of thalamus (25). Higher-order associative nucleuses are in the thalamic subregions, including the ventral anterior (VA) nuclei, ventral lateral posterior (VLp) nuclei, and most partial mediodorsal (MD) nuclei in the pre-frontal thalamus; the ventral lateral anterior (VLa) nuclei in the pre-motor thalamus; the ventral posterior lateral (VPL) nuclei in the sensory thalamus; the lateral posterior (LP) nuclei and partial pulvinar (Pu) nuclei in the parietal thalamus; the partial MD nuclei and partial Pu nuclei in the temporal thalamus, and the partial Pu nuclei in the occipital thalamus (25, 26). Abnormal thalamo-cortical FC was observed in previous studies. For example, Brown et al. found increased FC between medial thalamus and temporal areas, and between medial thalamus and somatosensory areas in MDD (27). Han et al. found increased and decreased FC between thalamic-subregions and cortex in long-term primary dysmenorrhea (28). Since thalamic subregions have different nucleuses and functions, examining the thalamo-cortical FC may promote our understanding of the pathophysiology of SD.

A number of studies have reported cognitive function deficits in patients with SD. Hall et al. found that physical complaints were correlated to poor performance in attention and psychomotor speed (29). Although the findings vary considerably across studies, there is an agreement that some domains of cognitive functions, such as memory (29–31), attention (29), executive function (29, 30), emotional awareness (32) and perceptual awareness (33) are impaired in patients with SD.

In addition, personality traits may be involved in the pathophysiological process of SD. A high prevalence of personality disorders in patients with SD has been reported about 20 years ago (34). Song et al. found a significantly positive correlation between the neuroticism scores of Eysenck Personality Questionnaire (EPQ) and increased regional homogeneity (ReHo) in the left angular gyrus (AG) in patients with SD (35). However, the relationship between cognitive function, personality, and thalamo-cortical FC remain unclear.

In the present study, we aimed to examine the thalamo-cortical FC in first-episode, treatment-naive patients with SD and conducted correlation analysis to explore the potential relationship between clinical symptoms, cognitive function, personality, and thalamo-cortical connectivity in SD. According to the Harvard Oxford subcortical structural atlases (25, 36), the thalamus can be subdivided into 7 bilateral subregions based on previous study (25). Based on previous studies (37, 38), we hypothesized that enhanced thalamo-cortical FC would exist in SD, and we also examined whether FC changes was positively correlated with cognitive function and personality traits in patients with SD.

## Methods

### Subjects

Twenty-six first-episode, drug-naive patients with SD from the Mental Health Center of the Second Affiliated Hospital, Guangxi Medical University in China from 2012.01 to 2013.12 were recruited, and 30 age-, sex-, and education-matched healthy controls from the local community were included. Each patient was diagnosed based on the Structured Clinical Interview of DSM-IV. The inclusion criteria for participants were as follows: Han Chinese ethnicity, 18 to 60 years old, drug-naive and right handedness. The exclusion criteria for patients with SD were as follows: any previous or current use of psychotropic medications, a history of neuropsychiatric disorders (comorbidity with depression is allowed because of the high comorbidity), and any contraindications for MRI scanning. The exclusion for healthy controls was as follows: no lifetime neuropsychiatric disorder, no family history of neuropsychiatric disorders, and no history of using of psychotropic drugs.

All participants were required to complete several questionnaires. Symptom severity was examined with Symptom checklist-90 (Scl-90) (39), Hamilton depression scale (HAMD; 17 items) (40) and Hamilton anxiety scale (HAMA) (41). Cognitive function was assessed by Wisconsin Card Sorting Test (WCST, including number of categories achieved, number of errors, and number of persistent error response of the test) (42) and digit symbol coding of Wechsler Adult Intelligence Scale (DSC-WAIS, including number of correctly decoded symbols in 90 seconds) (43). Personality traits were evaluated by EPQ (44), including four subscales (extraversion, neuroticism, psychoticism, and lie). These four subscales can represent positive affect/outgoing, negative affect/emotional unstableness, psychotic episode/aggression, and unsophisticated feature of an individual.

The present study was approved by the Ethics Committee of the Second Affiliated Hospital, Guangxi Medical University. All participants were given a complete description of the study and they provided a written informed consent.

### Image Acquisition

A 3.0 T Siemens scanner was used to collect data. All participants were required to lie motionless, eye-closed, and keeping quiet and awake for 500s during the image capturing. The MRI sequence type was an echo-planar imaging (EPI) sequence. The imaging-sequence parameters used were as follows: repetition time/echo time = 2000/30 ms, slices = 30, thickness= 4 mm, gap = 0.4 mm, field of view = 24 cm, flip angle= 90°, and data matrix = 64×64.

### Data Preprocessing

Data processing and analysis for (resting-state) brain imaging (45) was used to preprocess the entire MRI data.

The detailed procedure of FC analysis was as follows. First, slice timing and head motion were corrected. Subjects who had more than 2.0 mm maximum displacement in any direction (x, y, or z) and 2° of angular motion were excluded from the study. Second, the images were normalized to the standard Montreal Neurological Institute space in SPM8 with each voxel resampled to 3×3×3 mm^3^. Afterwards, the images were smoothed with a 4 mm full-width at half-maximum (FWHM) Gaussian kernel. The images were then temporally band-pass filtered (0.01–0.08 Hz) and linearly detrended. Moreover, sources of spurious covariates of head-motion parameters, cerebrospinal fluid signal, and white matter signal were removed. To address the residual effects of head motion, we computed the framewise displacement (FD) as described in the study of Power et al. (46) and used it as a covariate in group comparisons.

### FC Processing

Seven bilateral subregions of the thalamus from the Harvard Oxford subcortical structural atlases were selected as seeds for whole-brain FC processing with the REST software. The detail placement of each seed in direction (x, y, z) was shown in **Supplementary Material Figure S1**. The FC analysis was conducted as follows. First, to obtain a whole-brain FC matrix for each participant, Pearson correlation coefficients between the seeds and other voxels of the whole brain were calculated. Afterwards, the coefficients were standardized to *z*-scores to generate seed-based FC maps. Two sample *t*-tests were used to compare group differences, with HAMA scores, HAMD scores, age, sex, and the mean FD as covariates. The significance level was set at *p*<0.05 corrected by Gaussian random field (GRF) theory with a voxel threshold of *p*<0.001 and a cluster threshold of *p*<0.05(minimum cluster size=22 voxels).

### Statistical Analysis

Chi-square and *t*-tests were used to analyze the demographic characteristics. Correlation analyses between mean values of thalamo-cortical FC and clinical variables (including total questionnaire score and its factors, which reflect the severity of anxiety, depression and somatization symptoms, cognitive function, and personality dimensions) were performed by Pearson’s correlation analyses in patients with SD. Benjamini-Hochberg correction was used to limit type I error. Significance level was set at *p*<0.05.

## Results

### Demographics and Clinical Characteristics

We excluded data with excessive head movement, and then the data of 25 patients with SD and 28 healthy controls were analyzed. No significant difference was observed in terms of age, sex ratio, and education level. However, compared with healthy controls, patients with SD expressed higher scores in terms of HAMD scores, HAMA scores, somatization subscale of Scl-90, and EPQ psychoticism/neuroticism scores. Demographic information and clinical characteristics are shown in **Table 1**.

**Table 1:** Characteristics of participants.

Variables	Patients (n = 25)	Controls (= 28)	*p* value
Age (years)	41.00 ± 10.76	38.71 ± 9.59	0.42^b^
Sex (male/female)	4/21	6/22	0.73^a^
Years of education (years)	7.72 ± 4.39	7.82 ± 2.59	0.92^b^
Illness duration (months)	59.12 ± 62.22		
Somatization subscale of SCL-90	28.48 ± 10.37	14.32 ± 3.44	<0.001^b^
HAMD	18.84 ± 7.31	2.60 ± 1.83	<0.001^b^
HAMA	22.96 ± 10.95	0.53 ± 0.99	<0.001^b^
Digit symbol-coding of WAIS	8.28 ± 2.87	9.64 ± 2.15	0.06^b^
EPQ			
Extraversion	46.84 ± 11.02	49.75 ± 9.65	0.31^b^
Psychoticism	50.52 ± 9.01	45.00 ± 8.54	0.03^b^
Neuroticism	57.36 ± 9.18	46.78 ± 10.24	<0.001^b^
Lie	49.44 ± 12.31	47.96 ± 11.01	0.65^b^
WCST			
Number of categories achieved	3.52 ± 1.76	3.89 ± 1.66	0.43^b^
Number of errors	22.84 ± 9.12	24.71 ± 8.91	0.45^b^
WCST-Pre	20.04 ± 9.48	\22.82 ± 8.72	0.27^b^

a The p value for sex distribution was obtained by a chi-square test.

b The p values were obtained by two samples t-tests.

HAMD, Hamilton depression scale; HAMA, Hamilton anxiety scale; SCL-90, Symptom Checklist-90; EPQ, Eysenck Personality Questionnaire; WAIS, Wechsler Adult Intelligence Scale; WCST-Pre, persistent error response of Wisconsin Card Sorting Test.

### Seed-Based FC Analyses: Group Comparisons

**Figure 1:**
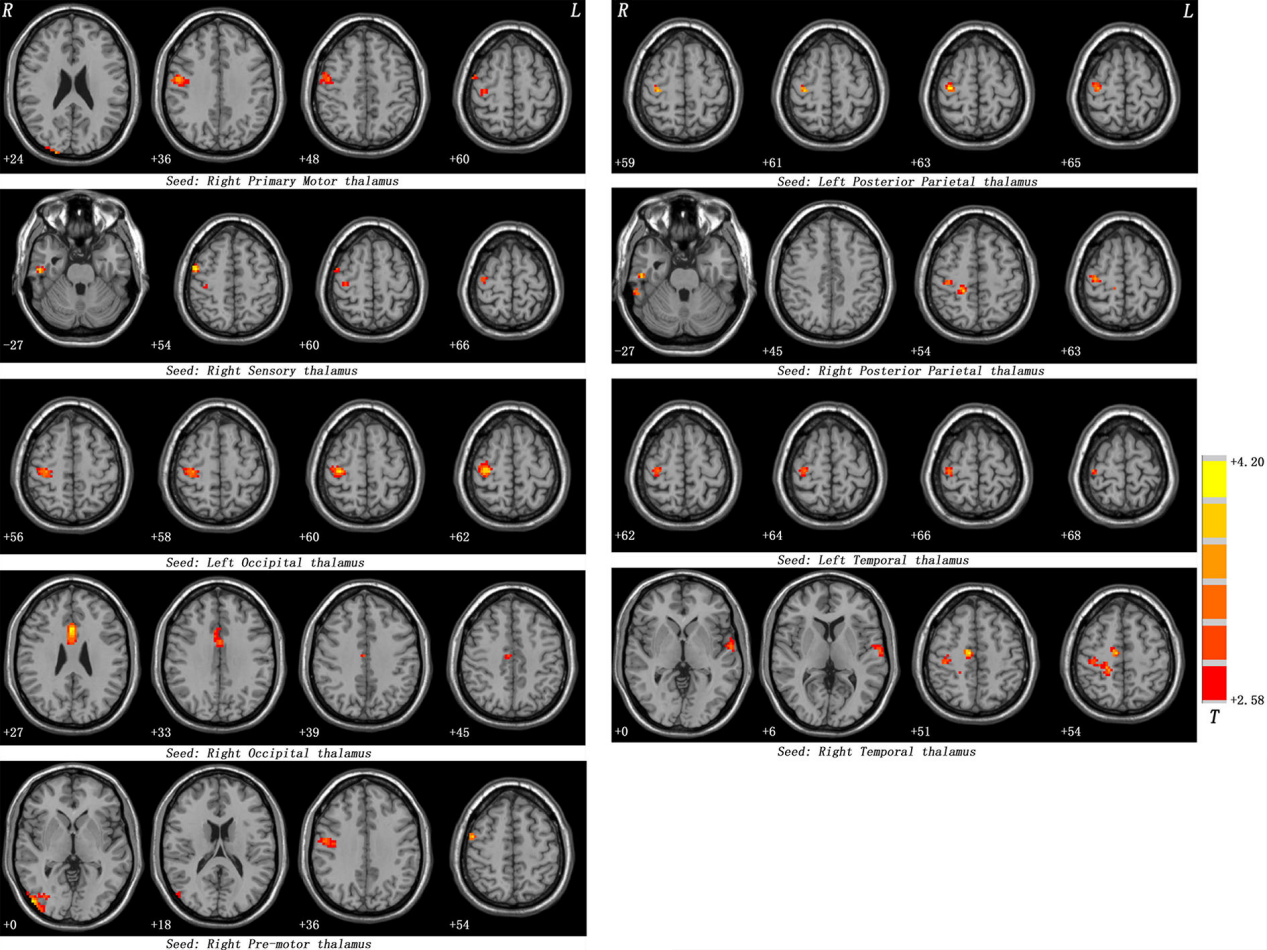
Maps of thalamo-cortical FC changes in patients with SD compared with healthy controls. Red denotes high FC values in patients and the color bar represents the *t* value from two-sample *t*-tests. Correction for multiple comparisons was conducted based on the Gaussian random field theory at *p *< 0.05 (voxel significance: *p *< 0.001, cluster significance: *p *< 0.05).

**Figure 2:**
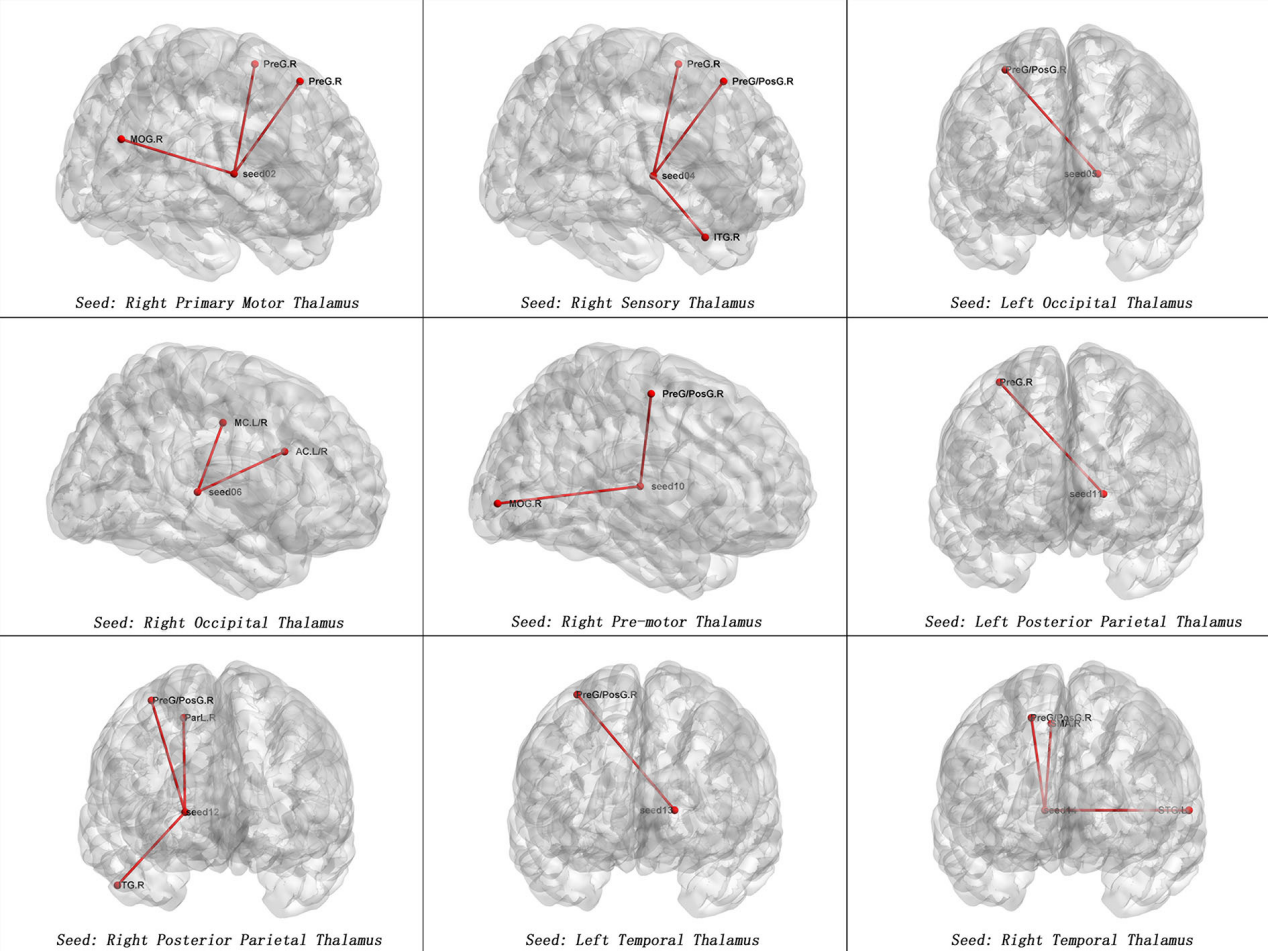
Maps of increased seed-based thalamo-cortical FC in patients with SD compared with healthy controls. The red line represents the significant increased FCs between subregions of thalamus and cortex. Correction for multiple comparisons was conducted based on the Gaussian random field theory at *p* < 0.05(voxel significance: *p *< 0.001, cluster significance: *p *< 0.05). MOG.R, right middle occipital gyrus, PreG.R, right precentral gyrus; ITG.R, right inferior temporal gyrus; PreG/PosG.R, right precentral gyrus/right postcentral gyrus; AC.L/R. anterior cingulum; MC.L/R, middle cingulum; ParL.R, right paracentral lobule; STG.L, left superior temporal gyrus, SMA.R = right supplementary motor area.

**Table 2** and **Figures 1**, **2** show hyperconnectivity between thalamic seeds and whole brain in patients with SD. Enhanced connectivity was found between the right primary motor thalamic ROI and the right middle occipital gyrus (MOG) and right precentral gyrus. We also found that right sensory thalamic ROI was positively connected with right precentral gyrus/right postcentral gyrus, the right ITG, and the right precentral gyrus. We observed significantly enhanced connectivity between the left occipital thalamic ROI and the right precentral gyrus/right postcentral gyrus, and between the right occipital thalamic ROI and the anterior cingulum and middle cingulum. Increased connectivity was found between the right pre-motor thalamic ROI and the right MOG and right precentral gyrus/right postcentral gyrus. The left posterior parietal thalamic ROI was positively connected with the right precentral gyrus, and the right posterior parietal thalamic ROI was positively connected with the right ITG, right precentral gyrus/right postcentral gyrus, and right paracentral lobule, respectively. Positive connectivity was also found between the left temporal thalamic ROI and the right precentral gyrus/right postcentral gyrus. The right temporal thalamic ROI was positively connected with the left superior temporal gyrus (STG), right precentral gyrus/right postcentral gyrus, and right SMA.

**Table 2:** Regions with increased connectivity with seeds in the patients.

Cluster location	Peak (MNI)			Number of voxels	*T* value
	x	y	z		
*Seed: left primary motor thalamus*					
None					
*Seed: right primary motor thalamus*					
Right middle occipital gyrus	21	−99	24	99	3.2895
Right precentral gyrus	51	−3	54	165	3.9532
Right precentral gyrus	36	−21	63	35	3.0798
*Seed: left sensory thalamus*					
None					
*Seed: right sensory thalamus*					
Right inferior temporal gyrus	54	−18	−27	29	3.464
Right precentral gyrus/right postcentral gyrus	51	−3	54	129	3.747
Right precentral gyrus	36	−21	63	27	3.0372
*Seed: Left occipital thalamus*					
Right precentral gyrus/right postcentral gyrus	33	−24	60	179	3.5828
*Seed: right occipital thalamus*					
Bilateral anterior cingulum	3	18	27	123	4.1951
Bilateral middle cingulum	6	−15	42	30	3.1134
*Seed: left pre-frontal thalamus*					
None					
*Seed: right pre-frontal thalamus*					
None					
*Seed: left pre-motor thalamus*					
None					
*Seed: right pre-motor thalamus*					
Right middle occipital gyrus	51	−84	0	200	4.1579
Right precentral gyrus/right postcentral gyrus	51	−3	57	122	3.7314
*Seed: left posterior parietal thalamus*					
Right Precentral Gyrus	36	−21	63	48	3.2562
*Seed: right posterior parietal thalamus*					
Right inferior temporal gyrus	54	−18	−33	62	3.4951
Right precentral gyrus/right postcentral gyrus	36	−21	63	57	3.1615
Right paracentral lobule	18	−36	54	28	3.2906
*Seed: left temporal thalamus*					
Right precentral gyrus/right postcentral gyrus	36	−24	66	57	3.0031
*Seed: Right Temporal thalamus*					
Left superior temporal gyrus	−63	−12	6	59	2.9629
Right precentral gyrus/right postcentral gyrus	21	−33	54	138	3.1737
Right supplementary motor area	9	−15	51	36	3.4222

The significance level was set at p < 0.05 corrected by Gaussian random field (GRF) theory (voxel significance: p < 0.001, cluster significance: p < 0.05, minimum cluster size = 22 voxels). HAMA scores, HAMD scores, sex, age, and the mean FD as covariates.

MNI, Montreal Neurological Institute; FD, framewise displacement.

### Correlations Between Increased FC and Clinical Variables in the Patients

The right primary motor thalamic ROI to the precentral gyrus was negatively correlated to scores of DSC-WAIS scores (*r* = −0.425, *p* = 0.034); the right sensory thalamic ROI to the right ITG was positively correlated to scl-90 somatic scores (*r*=0.407, *p*=0.043); the left occipital thalamic ROI to the right precentral gyrus/postcentral gyrus was negatively correlated to DSC-WAIS scores (*r* = −0.447, *p*=0.025); the right occipital thalamic ROI to the anterior cingulum was positively correlated to EPQ extraversion scores (*r*=0.404, *p*=0.045); the right occipital thalamic ROI to the anterior cingulum was positively correlated to HAMA total scores (*r*=0.397, *p*=0.050); the right occipital thalamic ROI to the middle cingulum was positively correlated to HAMA total scores (*r*=0.406, *p*=0.044); the right pre-motor thalamic ROI to the right precentral gyrus/postcentral gyrus was negatively correlated to DSC-WAIS scores (*r* = −0.470, *p*=0.018); the right posterior parietal thalamic ROI to the right ITG was positively correlated to scl-90 somatic scores (*r*=0.494, *p*=0.012); the left temporal thalamic ROI to the right precentral gyrus/postcentral gyrus was negatively correlated to DSC-WAIS scores (*r* = −0.540, *p*=0.005); and the right temporal thalamic ROI to the right precentral gyrus/postcentral gyrus was negatively correlated to DSC-WAIS scores (*r* = −0.427, *p*=0.033).

**Figure 3:**
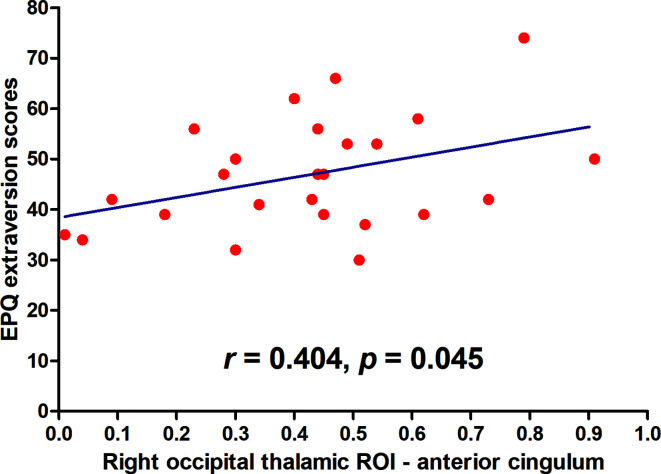
Maps of correlations between abnormal thalamo-cortical connectivity and personality traits. A significantly negative correlation was found between the right occipital thalamic ROI to the anterior cingulum and EPQ extraversion scores (*r* = 0.404, *p* = 0.045) after the Benjamini-Hochberg correction.

After the Benjamini-Hochberg correction, significant negative correlation was found between the right occipital thalamic ROI to the anterior cingulum and EPQ extraversion scores (*r*=0.404, *p*=0.045). See **Figure 3**.

## Discussion

We used thalamic subregions as seeds to conduct thalamo-cortical seed-based connectivity analysis and examine the specific thalamo-cortical FC in SD. To our knowledge, this report is the first on anatomically inferences of abnormal connectivity between thalamic subregions and cortex in patients with SD. The main findings were enhanced thalamic nuclei connectivity to the bilateral anterior/middle cingulum, motor/sensory cortex, visual cortex, and auditory cortex in patients with SD. These results suggested that patients with SD had increased thalamo-cortical FC with some specific regions which might be involved in the pathogenesis of SD. These findings may provide new insights into thalamo-cortical connectivity in patients with SD and help reveal the neurobiological mechanisms of SD.

One of the most important findings is the widespread increase in the thalamo-cortical network, including hyperconnectivity of the thalamic seeds to the bilateral anterior/middle cingulum, motor/sensory cortex, auditory cortex, and visual cortex. The findings of increased connectivity in the thalamo-cortical network may suggest a compensatory effort for either neuronal deficits or insufficient performance or nonselective recruitment occurring when appropriate regions have decreased accessibility (47). For example, Hua et al. found hyperconnectivity in the thalamus-prefrontal circuit, which implied a compensatory mechanism that additional cortical regions were recruited to subserve “normal” functions (48, 49). Moreover, given that the thalamo-cortical system modulates the transmission of sensory and motivation information, increased FCs reflect thalamic subregions hyper-responsiveness to negative emotions and cognitive function biased in patients with SD. We can infer that hyperconnectivity of the thalamus to the anterior/middle cingulum, motor/sensory cortex, auditory cortex, and visual cortex in the present study may be interpreted as a compensatory effort, a nonselective recruitment, or some combination of these two possibilities to reduce connectivity between thalamic nuclei and cortex in patients with SD.

Positive connectivity was found between the right occipital thalamic ROI and the anterior cingulum and middle cingulum. The occipital thalamus (Otha) includes Pu, which is involved in visual processing, attention, social cognition, and speech processing. Rafal et al. proposed a model for the posterior attention system, and found that patients with Pu lesions showed a deficit in the ability to hold attention on the target stimulus when competing information was also present in the visual field (50). The Pu was reported to act as an critical role in attentional modulation (51, 52). Zhou et al. provided causal information that Pu played an important role in attentive visual stimulus processing and maintaining and modulating neuronal oscillatory dynamics in visual cortex (53). Lemche et al. considered that the Pu was involved in separate brain system of depression (54). Furthermore, research considered that the Otha was related to the reward function (55). Li et al. found increased FC between the right occipital thalamus and right middle occipital gyrus, right amygdala, and right fusiform area, and decreased FC between the right Otha and left inferior parietal gyrus and left triangular inferior frontal gyrus in patients with obsessive-compulsive disorder (56). They suggested that the altered Otha-related FC was associated with reward processing, attention and emotion regulation (56). The anterior cingulum and middle cingulum play an important role in cognitive control by putting sensory input together to guide attention to salient stimuli and response selection through the recruitment of appropriate brain FC networks to modulate behavior. Dysfunction of anterior cingulum and middle cingulum may affect self-regulation of cognition, behavior, and emotion (57–59). In line with the results that the anterior cingulum and middle cingulum play a key role in cognitive control (60), researchers have reported that impaired cognition is widespread in patients with somatization symptoms (61, 62). Thus, we speculate that abnormal connectivity in Otha to the anterior/middle cingulum may play a potential role in imbalance of cognitive function in patients with SD. We also found a positive correlation between the connectivity of the right occipital thalamic ROI to the anterior cingulum and EPQ extraversion scores in the patients. The positive correlation suggests that a talkative patient has a strengthened connectivity of thalamic nuclei to the anterior cingulum cortex.

We found significantly increased connectivity between the right primary motor/sensory and left posterior parietal thalamic ROI and the right precentral gyrus; between the right sensory/pre-motor/posterior parietal/temporal and left occipital/temporal thalamic ROI and the right precentral gyrus/right postcentral gyrus; and between the right temporal thalamic ROI and the right SMA. The primary motor, sensory, pre-motor, posterior parietal, occipital, and temporal thalamus includes LP nucleus, VA nuclei, VLa nuclei, VPL nuclei, partial MD nuclei and partial Pu nuclei that project to the somatosensory cortices, temporal lobe, pre-motor areas, primary motor cortex and posterior parietal lobe (25). These nucleuses were involved in sensory and motivation information transmutation. Patients with SD pay overfull concentration on physical sensations, that leads to a decrease in awareness of events in the external world (63, 64), resulting in an imbalance between internal and external stimuli (21). Therefore, impaired top-down and bottom-up modulations of sensory and motivation exist in SD (65, 66). The motor/sensory cortex, including somatosensory (postcentral gyrus), motor (precentral gyrus), and SMA (67), is specialized in processing of sensory stimuli and motor responses. Studies have reported that the motor/sensory cortex plays an important role in bipolar disorder (68, 69), suggesting the potential compensatory effort of the motor/sensory cortex in emotional regulation. Patients with SD have unexplained physical complaints leading to frequently visit hospitals for physical examinations (70). Thus, increased thalamic FC with motor/sensory cortex shown in the present study partially explains specific behaviors in patients with SD.

Furthermore, we observed that hyperconnectivity existed between the right temporal thalamic ROI and the left STG; between the right sensory/right posterior parietal thalamic ROI and the right ITG; and between the right premotor thalamic ROI and the right MOG in the patients. These findings suggested hyperconnectivity in thalamus nuclei to the auditory and visual cortex. The STG and ITG are responsible for processing sounds. As essential structures involved in auditory processing, including language, the STG and ITG are also associated with social cognition processes (71, 72). The MOG, which is located in the occipital lobe, participates in visual processing (73). These increased FCs in thalamus nuclei to the auditory and visual cortex further support the disturbance of somatosensory processing and the motor/sensory cortex.

In the present study, patients with SD scored higher in HAMA and HAMD than healthy controls. To reduce possible effects of HAMA and HAMD scores on thalamo-cortical FC, we used age, sex, the mean FD, HAMA scores and HAMD scores as covariates in the analyses. In addition, we used age, sex and the mean FD (without HAMA and HAMD scores) as covariates to reanalyze the data, and obtained similar results (**Table 2** and **Supplementary Material Table S1**). This issue indicates that HAMA and HAMD scores have little effects on the present results, which suggests that abnormal thalamo-cortical FC may be an inherent characteristic for SD that can be used as a potential endophenotype for SD.

Limitations exist in the study. First, the relatively small sample cannot be ignored. Second, this study is based on resting-state fMRI, so we cannot eliminate physiologic noise. Third, we found high levels of anxiety and depression symptom severity in the patients. A high ratio of comorbidities is known to exist among depression, anxiety, and somatization (74). Since anxiety and depression may be inherent characteristics of SD, the influence cannot be completely removed in the analysis. Given the imbalanced sex ratio (female-to-male ratio= 5:1) in the general population, we recruited high proportion of females in the study.

In conclusion, our findings demonstrated increased FC between the thalamic subregions and cortex in patients with SD. Increased connectivity in thalamus nuclei to the anterior/middle cingulum and motor/sensory cortex were observed, indicating the important role of disturbed functional networks in the pathophysiology of SD. Overall, our study highlights the importance of the thalamic subregions in the pathophysiology of SD.

## Data Availability Statement

All datasets presented in this study are included in the article/**Supplementary Material**.

## Ethics Statement

The studies involving human participants were reviewed and approved by The Ethics Committee of the First Affiliated Hospital, Guangxi Medical University. The patients/participants provided their written informed consent to participate in this study. Written informed consent was obtained from the individual(s) for the publication of any potentially identifiable images or data included in this article.

## Author Contributions

WG and JPZ designed the study. JZ and QS collected the original imaging data. FL, RY, and WG managed and analyzed the imaging data. JZ and ZZ wrote the first draft of the manuscript.

## Funding

This study was supported by grants from the National Key R&D Program of China (grant nos. 2016YFC1307100 and 2016YFC1306900), the National Natural Science Foundation of China (grant nos. 81771447 and 81630033), and the Natural Science Foundation of Tianjin (grant no. 18JCQNJC10900).

## Conflict of Interest

The authors declare that the research was conducted in the absence of any commercial or financial relationships that could be construed as a potential conflict of interest.
