# Supplementary material for: Enhanced Connectivity of Thalamo-Cortical Networks in First-Episode, Treatment-Naive Somatization Disorder
Source: Front Psychiatry. 2020 Sep 11;11:555836. doi: 10.3389/fpsyt.2020.555836 (PMC7518236; doi:10.3389/fpsyt.2020.555836)
Supplement: Supplementary file 1 [file DataSheet_1.docx]

Table S1. Regions with increased connectivity with seeds in the patients.

| Cluster location | Peak (MNI) | | | Number of voxels | *T* value |
| --- | --- | --- | --- | --- | --- |
|  | x | y | z |  |  |
|  |  |  |  |  |  |
| *Seed: Left Primary Motor thalamus -18，-20,6* |  |  |  |  |  |
| None |  |  |  |  |  |
| *Seed: Right Primary Motor thalamus 18，-20,6* |  |  |  |  |  |
| Right Middle Occipital Gyrus | 21 | -99 | 24 | 95 | 3.2893 |
| Right Precentral Gyrus | 51 | -3 | 54 | 151 | 3.9237 |
| Right Precentral Cyrus | 36 | -21 | 63 | 28 | 2.9911 |
| *Seed: Left Sensory thalamus -18,-23,5* |  |  |  |  |  |
| None |  |  |  |  |  |
| *Seed: Right Sensory thalamus 18,-23,5* |  |  |  |  |  |
| Right Inferior Temporal Gyrus | 54 | -18 | -27 | 28 | 3.4388 |
| Right Precentral Gyrus/ Right Postcentral Gyrus | 51 | -3 | 54 | 115 | 3.6921 |
| Right Precentral Gyrus | 36 | -21 | 63 | 24 | 3.3156 |
| *Seed: Left Occipital thalamus -15,-30,6* |  |  |  |  |  |
| Right Precentral Gyrus/Right Postcentral Gyrus | 33 | -21 | 63 | 202 | 3.8270 |
| *Seed: Right Occipital thalamus 15,-30,6* |  |  |  |  |  |
| Bilateral Anterior Cingulum | 3 | 18 | 27 | 109 | 4.1008 |
| Bilateral Middle Cingulum | 6 | -15 | 42 | 29 | 2.9819 |
| *Seed: Left Pre-frontal thalamus -9,-14,6* |  |  |  |  |  |
| None |  |  |  |  |  |
| *Seed: Right Pre-frontal thalamus 9,-14,6* |  |  |  |  |  |
| None |  |  |  |  |  |
| *Seed: Left Pre-motor thalamus -16,-15,9* |  |  |  |  |  |
| None |  |  |  |  |  |
| *Seed: Right Pre-motor thalamus 16,-15,9* |  |  |  |  |  |
| Right Middle Occipital Gyrus | 51 | -84 | 0 | 190 | 4.1103 |
| Right Precentral Gyrus/Right Postcentral Gyrus | 51 | -3 | 54 | 119 | 3.6668 |
| *Seed: Left Posterior Parietal thalamus -18,-27,5* |  |  |  |  |  |
| Right Precentral Gyrus | 33 | -21 | 63 | 50 | 3.3027 |
| *Seed: Right Posterior Parietal thalamus 18,-27,5* |  |  |  |  |  |
| Right Inferior Temporal Gyrus | 54 | -18 | -33 | 33 | 3.4769 |
| Right Precentral Gyrus/Right Postcentral Gyrus | 36 | -21 | 63 | 39 | 3.0361 |
| Right Paracentral Lobule | 18 | -36 | 54 | 27 | 3.3624 |
| *Seed: Left Temporal thalamus -7,-18,17* |  |  |  |  |  |
| Right Precentral Gyrus/Right Postcentral Gyrus | 33 | -24 | 66 | 95 | 3.1897 |
| *Seed: Right Temporal thalamus 7,-18,17* |  |  |  |  |  |
| Left Superior Temporal Gyrus | -63 | -12 | 6 | 43 | 2.9166 |
| Right Precentral Gyrus/Right Postcentral Gyrus | 21 | -33 | 54 | 167 | 3.1565 |
| Right Supplementary Motor Area | 9 | -15 | 51 | 44 | 3.4261 |

The significance level was set at *p*< 0.05 corrected by Gaussian random field (GRF) theory (voxel significance: *p*<0.001, cluster significance: *p*<0.05, minimum cluster size=22 voxels). Sex, age and the mean FD as covariates.

MNI = Montreal Neurological Institute; FD=framewise displacement

Figure S1: Subregions of the thalamus in a T1 image. The voxel sizes of the subregions of the thalamus were as follows (1 voxel = 3×3×3 mm3): Left Primary Motor thalamus: 52 voxels; Right Primary Motor thalamus: 64 voxels; Left Sensory thalamus: 44 voxels; Right Sensory thalamus: 73 voxels; Left Occipital thalamus: 116 voxels; Right Occipital thalamus: 53 voxels; Left Pre-frontal thalamus: 288 voxels; Right Pre-frontal thalamus: 248 voxels; Left Pre-motor thalamus: 85 voxels; Right Pre-motor thalamus: 99 voxels; Left Posterior Parietal thalamus: 153 voxels; Right Posterior Parietal thalamus: 152 voxels; Left Temporal thalamus: 249 voxels; Right Temporal thalamus: 258 voxels.
